# Unveiling cross-reactivity: implications for immune response modulation in cancer

**DOI:** 10.1093/bib/bbaf012

**Published:** 2025-01-20

**Authors:** Marco Antônio M Pretti, Gustavo Fioravanti Vieira, Mariana Boroni, Martín H Bonamino

**Affiliations:** Laboratory of Bioinformatics and Computational Biology, Division of Experimental and Translational Research, Brazilian National Cancer Institute (INCA), Rio de Janeiro, Brazil; Program of Cell and Gene Therapy, Division of Experimental and Translational Research, Brazilian National Cancer Institute (INCA), Rio de Janeiro, Brazil; Postgraduate Program in Genetics and Molecular Biology, UFRGS, Porto Alegre, Brazil; Postgraduate Program in Health and Human Development, La Salle University, Canoas, Brazil; Laboratory of Bioinformatics and Computational Biology, Division of Experimental and Translational Research, Brazilian National Cancer Institute (INCA), Rio de Janeiro, Brazil; Program of Cell and Gene Therapy, Division of Experimental and Translational Research, Brazilian National Cancer Institute (INCA), Rio de Janeiro, Brazil; Vice-Presidency of Research and Biological Collections (VPPCB), Oswaldo Cruz Foundation (FIOCRUZ), Rio de Janeiro, Brazil

**Keywords:** cross-reactivity, T-cell receptor, molecular docking, HLA, cancer, virus

## Abstract

Antigen recognition by CD8^+^ T-cell receptors (TCR) is crucial for immune responses to pathogens and tumors. TCRs are cross-reactive, a single TCR can recognize multiple peptide-Human Leukocyte Antigen (HLA) complexes. The study of cross-reactivity can support the development of therapies focusing on immune modulation, such as the expansion of pre-existing T-cell clones to fight pathogens and tumors. The peptide-HLA (pHLA) surface has previously been used to identify TCR cross-reactivities. In the present work, we sought to perform a comprehensive analysis of peptide-HLA by selecting thousands of human and viral epitopes. We profit from established docking models to identify features from different spatial perspectives of HLA-A^*^02:01, explore similarities between self and non-self epitopes, and list potential cross-reactive epitopes of therapeutic interest. A total of 2631 unique epitopes from representative viral proteins or human proteins were modeled. We were able to demonstrate that cross-reactive CDR3 sequences from public databases recognize epitopes with similar electrostatic potential, charge, and spatial location. Using data from published studies that measured T-cell reactivity to mutated epitopes, we observed a negative correlation between epitope dissimilarity and T-cell activation. Most analysed cancer epitopes were more similar to self epitopes, yet we identified features distinguishing those more similar to viral antigens. Finally, we enumerated potential cross-reactivities between tumoral and viral epitopes and highlighted some challenges in their identification for therapeutic use. Moreover, the thousands of peptide-HLA complexes generated in our work constitute a valuable resource to study T-cell cross-reactivity.

## Introduction

Antigen recognition by T-cells is at the core of immune responses to pathogens and tumors [1]. In this sense, the interaction between Human Leukocyte Antigen (HLA) complexes presenting foreign peptides (pHLA) and T-cell receptors (TCRs) activates the effector functions of the latter [2]. TCRs are generated through gene rearrangement which produces a diverse repertoire estimated to be around 10^12^, with a higher diversity found at the complementary determining regions (CDR), especially the CDR3 of beta chains [3]. Despite the diverse repertoire and specificity, T-cell cross-reactivity is needed to offer a comprehensive protection against pathogens. Mason was among the first to propose it in 1998 [4], which was further demonstrated [3, 5–10] in both mice [7] and humans [6, 8]. TCRs may interact with up to 10^6^ different epitopes [11] and with different affinities [12], which affects the level of T-cell activation [13] and consequently cytokine release by the T-cell [14, 15]. Several studies have investigated the extent and structural basis for TCR cross-reactivity [5, 7, 8, 14, 16, 17]. For instance, Birnbaum et al.[7] profited from deep sequencing and identified amino acid positions within the TCR-pHLA interaction related to TCR cross-reactivity [7]. Later on, Riley et al. [18] presented a case in which TCR-pHLA interaction provoked dramatic changes in the peptide conformation. Previous works described the use of pHLA structures to infer cross-reactivity in hepatitis C virus [19], and recently between BCG and SARS-CoV-2 [20]. They used the electrostatic potential on the pHLA surface as a proxy for TCR cross-reactivity [21, 22], which has implications for cancer and autoimmunity [23]. Autoreactive T-cells might become activated during life by recognizing epitopes similar to self antigens, a process known as molecular mimicry. In fact, this has been suggested as a probable cause of some autoimmune diseases [24, 25]. Conversely, cancer immunotherapy aims to eliminate tumor cells that express antigens highly similar to self-antigens. In this sense, the expansion of cross-reactive T-cell clones reactive to pathogens and cancer antigens might be of therapeutic interest [26, 27]. Therefore, understanding the biology behind TCR cross-reactivity would help (i) the efficient design of T-cell vaccines that account for escape variants [21, 28]; (ii) the prediction and identification of cross-reactivity to self antigens [14] that might help manage autoimmune diseases; (iii) the expansion of pre-existing tumor-reactive T-cells [29], among others. In the present work, we showed that cross-reactive HLA-A2-restricted epitopes are mostly distinguished from a pool of representative and non-related epitopes based on pHLA charge and electrostatic potential. We associated T-cell activation and electrostatic potential and charge to recapitulate TCR reactivity. Finally, we investigated pHLA similarity between viral and human epitopes that could be at the origin of cross-reactivities.

## Materials and methods

### Selection of epitopes and CDR3 sequences

VDJdb [30], McPAS [31], and TBAdb [32] databases containing epitope and TCR sequences of *in vitro* T-cell assays were queried in October 2022. VDJdb was filtered for entries matching “species = Human, TR = TRA or TRB, Class = MHC-I”. Similarly, McPAS and TBAdb were filtered using “*Homo Sapiens*”, “TRA-TRB”, “TRA”, “TRB”. Only nonamers presented by alleles annotated as HLA-A^*^02:01 or HLA-A^*^02 were kept. CDR3 sequences from alpha and beta chains that recognize more than one epitope were annotated as cross-reactive and used to select epitopes for docking. Representative peptides from viral proteins were obtained from the National Center for Biotechnology Information (NCBI) database by searching for “Identical Protein groups”‘using the keywords “Viruses[Organism] AND srcdb_refseq[PROP] NOT cellular organisms[ORGN]”. Sequences annotated as putative were excluded with seqkit 2.0.0. The remaining viral proteins were used to predict binding to HLA-A^*^02:01 with netMHCpan4.1 as previously described [33]. Peptides were ranked and 1000 binders with the lowest %Rank were selected. Similarly, binding predictions for human proteome GRCh38 were performed to select 1000 peptides. Studies testing TCR reactivity in vitro have been reviewed, and selected peptides were modeled [14, 15, 34–36]. Decamers from Bulek and collaborators were used in binding predictions as described above to obtain the nonamer binding core.

### HLA-A^*^02:01 docking and calculation of electrostatic potential

Docking of selected peptides in the HLA-A^*^02:01 (PDB ID: 2GTW) was performed with Docktope [37] implemented in the high-performance computing cluster within the singularity container. PDB files generated by DockTope were converted to PQR format with PDB2PQR using AMBER force field before calculating the electrostatic potential with APBS method (Adaptive Poisson-Boltzmann Solver) [38]. Electrostatic potentials of modeled pHLA were loaded into Pymol [39] and colored to represent surface charge distribution using a scale of-10/+10kT. Top view of the pHLA was obtained by rotating the molecule (x-axis 60°, z-axis-80°, y-axis-10°, and x-axis-10°). Four other perspectives were captured by rotating-10° or + 10° in the x and y-axis from the ‘top’ perspective.

### Data structuring

Modeled pHLAs contain information regarding the number and name of the atom, residue, spatial localization (XYZ), charge, and radius. They were annotated to identify the β2-microglobulin, HLA chains, and epitope. To ensure uniformity in the number of entries (atoms), atoms from each residue and chain (including B2M, HLA, and the epitope) were aggregated. This was achieved by averaging the charge, radius, and spatial coordinates. Moreover, residues from the epitope and extracellular portion of the HLA (residue number ≤ 180) were selected, excluding the β2-microglobulin and intracellular residues. Finally, all modeled epitopes presented the same number of entries depending on the filtering: 384 (pHLA), 189 (extracellular portion of pHLA, ec pHLA) or 9 (epitope).

### pHLA image processing

Images modeled pHLAs were imported into R using the png v0.1–8 package and decomposed into RGB matrices. Each image generated four 640 x 480 matrices corresponding to the intensities of red, green, blue, and transparency. In order to reduce data size and complexity, the following operation was performed to obtain a single matrix per epitope: red - blue + transparency. In this sense, the differences between the negative (red) and positive (blue) extremes of the electrostatic potential are highlighted. To identify the most informative regions of pHLA, the standard deviation of images having viral or human epitopes was calculated. To avoid introducing bias from sequence similarity, synthetic epitopes were excluded. Pixels with a standard deviation greater than the median were selected. Next, the central region of pHLA was selected to exclude regions with high standard deviation but absent from the contact zone with the TCR. The intersection between the central region of the image and areas with high standard deviation was used.

### Statistical analysis

Dimensionality reduction was performed on the resultant matrix of pHLA images. Uniform Manifold Approximation and Projection (UMAP) projections were obtained using functions from Seurat v4.0.0. For the structured data and pHLA images, distances between pHLA matrices were calculated using Euclidean and Canberra distances, respectively. Kruskal-Wallis was performed for comparisons with more than two groups using ggpubr v0.6, Spearman correlations were calculated using the Hmisc v4.4, Cohen’s effect size, and Wilcoxon test with rstatix v0.7.2. Percent sequence identities were calculated with the pid function using type = ‘PID1’ from Biostrings v2.68. Figures were generated in R using ggplot2 v3.3.1, ggseqlogo v0.1, cowplot v1.0.0, pheatmap v1.0.12, ggbreak v0.1.1, and RColorBrewer v1.1. Other relevant packages used throughout the analysis were data.table v1.13.0, dplyr v1.0 and tibble v3.0.1. All statistical analyses were performed in R v4.0.5.

## RESULTS

### Cross-reactive CDR3 recognizes epitopes with similar pHLA electrostatic potential, charge, and spatial position

**Figure 1:**
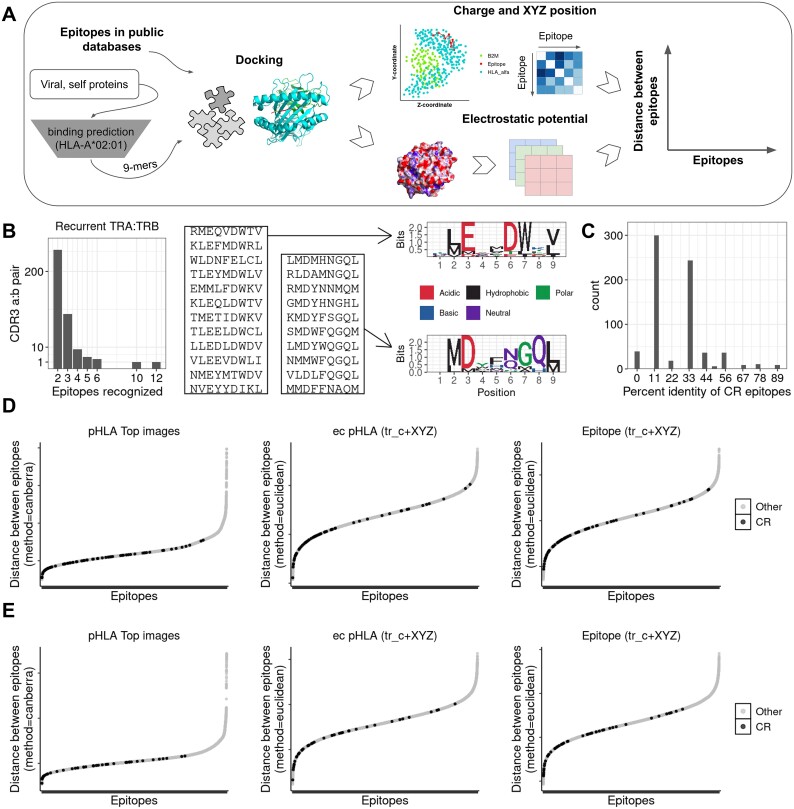
Cross-reactive CDR3s recognize epitopes with similar pHLA electrostatic potential, charge, and spatial position. A) HLA-A^*^02–01-restricted epitopes described in public databases were modeled using DockTope yielding 987 viral and 989 self epitopes. The charge and spatial position of docked epitopes and the more extracellular portion of the pHLA (ec pHLA) were obtained to calculate the distance between them. Similarly, the images of the electrostatic potentials were decomposed into color matrices to calculate the distance between pHLA (see methods). B) Number of CDR3 alpha (TRA) and beta (TRB) associated with at least two epitopes. Sequence of the epitopes recognized by the two most cross-reactive TCRs in the public database, recognizing 12 or 10 different epitopes. On the right, the logo plot illustrates the conserved residues at each position (height is associated with frequency) and their properties. C) Sequence similarity of epitopes recognized by cross-reactive (CR) CDR3. Epitopes associated with each CDR3 were selected to calculate the percent sequence identities between them. If three or more epitopes were associated with a given CR CDR3, the sequence identity was calculated for each combination. A score of 100 represents a perfect match between the sequences while 0 means no match. D-E) distance between epitopes calculated from the electrostatic potential of pHLAs or charge and spatial position without anchor positions (tr_c + XYZ). In the representation of epitope pairs, those recognized by cross-reactive (CR) TCRs are depicted in black, whereas the remaining epitopes are shown in gray. This distinction is made for both sets of 12 epitopes (D) and 10 epitopes (E).

To predict TCR reactivity and cross-reactivity, we used a large-scale approach to associate the electrostatic potential of pHLA complexes with CDR3 sequences recognizing them. For that purpose, public databases [30–32] and published papers describing mutated epitopes [14, 15] were assessed to retrieve experimental assays of nonamers presented through HLA-A^*^02:01. From the databases, we selected CDR3 beta sequences that recognize at least two nonamers, termed here as cross-reactive CDR3 (Supplementary Data S1). To consider the varying charge patterns of epitopes not present in public databases, binding predictions to HLA-A^*^02:01 were performed for all peptides from the human proteome and 176 991 representative viral proteins from the NCBI database, as shown in Fig. 1A. Afterward, this second set of epitopes allowed the comparison between viral and human epitopes. Prior to docking, 1000 human peptides or viral peptides were selected yielding 989 self and 987 viral epitopes.

In total, 2631 peptides (Table S1, Supplementary Data S1) were successfully modeled with DockTope [37]. The pHLA produced was then used to calculate the electrostatic potential at the surface of the molecule and screenshots from five perspectives were obtained to account for slight changes in the TCR engagement angle (Fig. S1, Fig. 1A). As depicted for three epitopes derived from MAGEA11, Gag Polyprotein, and pre-pro-insulin (PPI), slight changes in color and structure are observed across the three pHLA. Moreover, rotation of the MAGEA11 pHLA evidences previously unseen regions of the molecule. The use of multiple perspectives alone or combined could better reflect changes in the pHLA and improve the prediction of cross-reactive responses.

Of the 15 339 CDR3 alfa/beta pairs described in the public databases, 369 were associated with multiple epitopes (Fig. 1B). We selected the epitopes that were recognized by two of these promiscuous TCRs, recognizing 10 (TRB:CASSRDTVNTEAFF; TRA:CALSEARGGATNKLIF) or 12 epitopes (TRB:CASSPSGLAGSNLGNEQFF; TRA:CALSSRGSTLGRLYF), and calculated the distance among the epitopes recognized by them and the other modeled epitopes. These epitopes present rather conserved motifs at positions 3, 6, and 7, regions contacting the TCR (Fig. 1B). As expected, HLA anchor positions P2 and P9 have conserved residues. However, most epitopes recognized by cross-reactive CDR3 from the databases have sequence identities of one or three residues (Fig. 1C). The electrostatic potential, charge, and spatial position were used to calculate the distances between epitopes (Fig. 1D). We expect that lower distances reflect similarities between epitopes and thus recapitulate the observed cross-reactivities of the promiscuous CDR3. Shorter distances were observed for the 12 epitopes recognized by the cross-reactive CDR3 when compared to other epitopes, either in the pHLA images (median 2012 versus 2333, *P* = 5.15e-17), the ec pHLA, (3.93 versus 4.81, *P* = 3.19e-30), and epitopes (3.25 versus 4.18, *P* = 5.94e-26). Similar results were found for the cross-reactive CDR3 recognizing 10 epitopes in both pHLA images (1888 versus 2082, *P* = 8.11e-14), ec pHLA (4.33 versus 5.19, *P* = 1.99e-18), and epitope charge and spatial positions (3.81 versus 4.63, *P* = 5.62e-18, Fig. 1E). Both electrostatic potential and charge were able to separate most epitopes recognized by cross-reactive CDR3 from a pool of unrelated epitopes.

**Figure 2:**
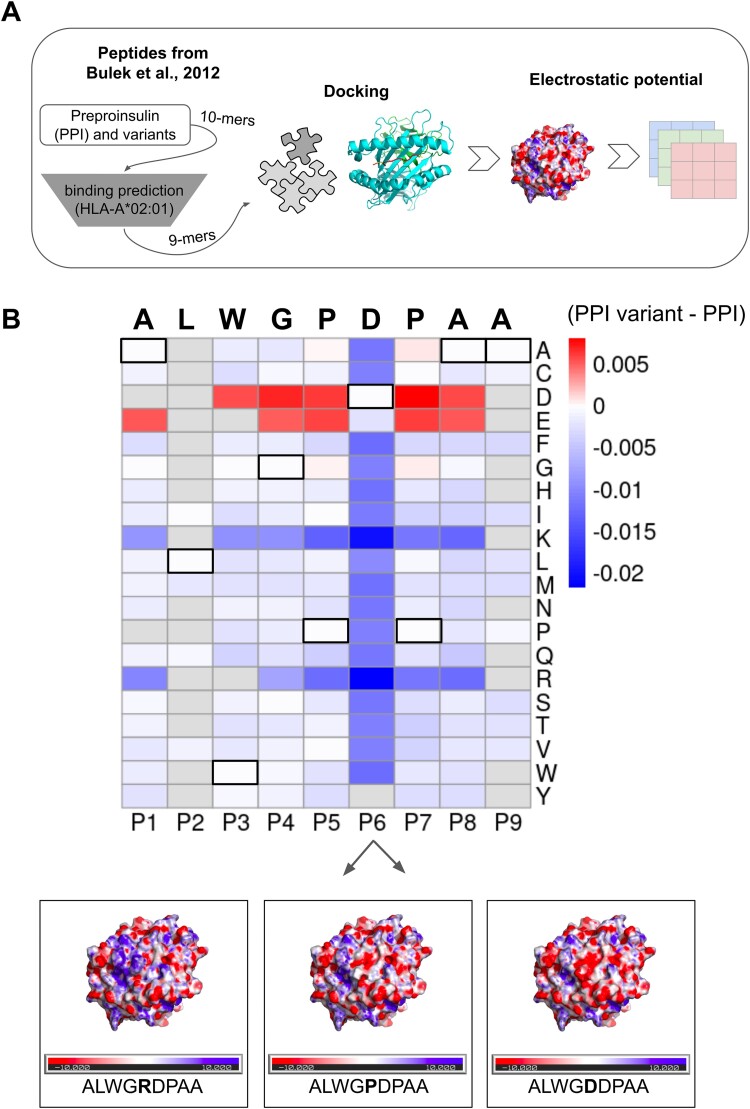
Electrostatic profile of Preproinsulin variants. A) Workflow for obtaining the images of electrostatic potential. All variants of the PPI ALWGPDPAA epitope were used for binding predictions to HLA-A^*^02:01 to select the core nonamers to be modeled by Docktope. The electrostatic potential was calculated from the modeled structures as described in the methods and the colors of the images were divided into layers of red, green, and blue prior to manipulation and clustering. B) Heatmap of electrostatic potential changes in the pHLA compared to the PPI epitope. For the 143 variant epitopes, the color difference to the PPI epitope was plotted to represent the gain of negative (red) or positive charge (blue). Black squares outline the PPI sequence at the top. Residues absent at a specific position are shown in gray. At the bottom, three of the modeled complexes are represented, with a negatively charged residue at position P6 (right), the original epitope (center), and a positively charged residue at position P6 (left). Blue and white colors represent positive and neutral charges, respectively.

Previous works described the relationship between the observed electrostatic potential and the amino acid charge [22]. In this sense, we sought to explore this association by leveraging position-mutated epitopes derived from a PPI epitope, a recognized epitope in type-1 diabetes [14]. This set of peptides provides a good benchmark to assess TCR reactivity since changes in the sequence impact the immunogenicity. Binding predictions of PPI and 199 variants were performed to filter for binders and select the nonamer core to be modeled (Fig. 2A). The electrostatic potential of mutated epitopes (n = 143) was subtracted from the PPI to depict the gain (red) or loss (blue) of negative charge (Fig. 2B). HLA anchor positions P2 and P9 were determinants in the selection of binder peptides since most residues yield no change at these positions (gray color). Most changes were generated by acid (D and E) and basic (K and R) residues, slightly impacting the surrounding residues. This is exemplified by the three structures at the bottom which possess a basic residue (left), the original (center), or acid (right). Besides the electrostatic potential, we also explored the charge and spatial position of the residues for the PPI and its variants. We observed that most PPI variants presented lower charge in the residues P3, and P5 to P7 (Fig. S2A) and higher freedom in the central residues, especially in P7 (Fig. S2B). As expected, anchor positions P2 and P9 presented more spatial constraints which reflected in a lower dispersion index. To confirm that, we repeated the same approach on all modeled pHLA without the synthetic peptides (n = 2304). A similar trend was observed for the charge and spatial position of the residues (Fig. S2C-D). Besides, the increased number of epitopes provided a higher resolution in an unbiased manner. In both cases, the variation in charge between the peptides was higher in the P6 and P4 and lower in the P9. Those changes were also reflected in the electrostatic potential, as observed by the higher variation in color on the peptide region of different pHLA perspectives (Fig. S3). Together, the findings confirm that the modeling and downstream analysis of charge, spatial localization, and electrostatic potential produce results compatible with those reported in the literature. Moreover, they highlight the huge impact that acid and basic residues have on the electrostatic potential of pHLA.

### Electrostatic charges of pHLA partially recapitulate TCR reactivity

**Figure 3:**
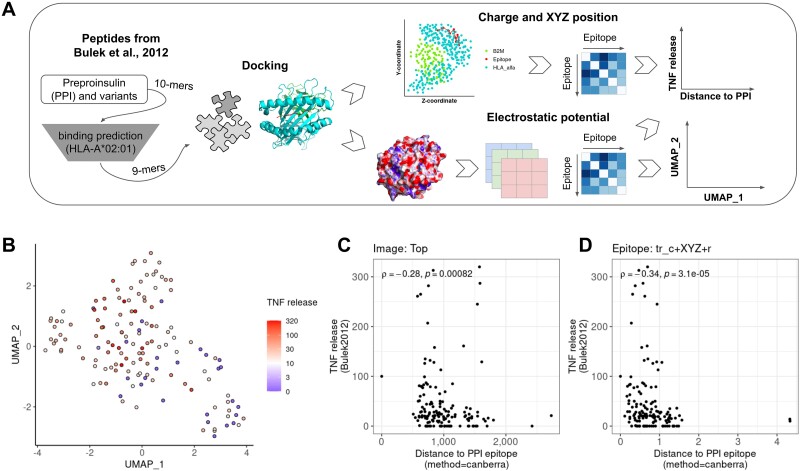
Association of TCR cross-reactivity with TNF release. A) Workflow for obtaining the images of electrostatic potential. All variants of the PPI ALWGPDPAA epitope were used for binding predictions to HLA-A^*^02:01 to select the core nonamers to be modeled by Docktope. The electrostatic potential was calculated from the modeled structures as described in the methods and the colors of the images were divided into layers of red, green, and blue prior to manipulation and clustering. The charge and spatial position of each modeled structure were also obtained to calculate the Euclidean distances between epitopes and further used to test the correlation with the TCR reactivity. B) UMAP visualization of the electrostatic profile images of peptide-HLA complexes for the 144 selected epitopes from Bulek et al. after dimensionality reduction. The images were obtained from five perspectives that encompass the HLA groove, and pixels were selected for regions of higher variability (see methods). C-D) Spearman correlation between the similarity of variant epitopes of the PPI and TNF-⍺ release. The Canberra distance was used to calculate the distance between the epitope images and the PPI (C), and the Euclidean distance for the distance between charge and spatial position between the epitopes and the PPI (D).

One of the biggest challenges in T-cell reactivity is finding determinants that explain the cross-reactivity triggering TCRs. Here, by using *in vitro* data generated by Bulek *et al.* [14], we associated T-cell activation measured by TNF release with the *in silico* findings. Images of electrostatic potential were decomposed as previously described (Fig. 3A) and unsupervised clustering was performed on the matrices to spot associations between spatial segregation and TNF release (Fig. 3B). The UMAP shows that the epitopes on the bottom right have lower TNF release. A similar pattern was also observed for the other pHLA perspectives (Fig. S4A). TNF release correlated with the first (Pearson’s R = −0.21, *P* = 0.0132) and second UMAP dimensions (Pearson’s R = 0.18, *P* = 0.0286). Multiple linear regression showed a significant association between the two UMAP dimensions and TNF release (*P* = 0.02185). In a different approach, we performed Spearman correlations to investigate whether the distance between the images of the PPI epitope and its variants is associated with the TNF release. In this sense, variants closer to zero present similar images to the PPI epitope than images with higher values. A weak correlation (rho = −0.28, *P* = 0.00082) was observed for the pHLA (top perspective) suggesting that epitopes less related to PPI trigger a lower TNF release by the T-cell clone (Fig. 3C). Similarly, correlations were performed using the median charge and spatial position of the epitope yielding weak correlations (rho = −0.34, *P* = 3.1e-05) with the TNF release (Fig. 3D). A summary of all correlations is depicted in Fig. S4B, comprising a control of amino acid properties described by Atchley [40] charge and spatial coordinates of pHLA molecules, the ec pHLA or the epitope; the electrostatic potential of pHLA alone, in combination with other perspectives or combined with the charge and spatial position. We observed that the strongest correlations included the charge and spatial position of the epitope alone or in combination with the electrostatic potential of the pHLA. Surprisingly, combining multiple perspectives of the electrostatic potential (all perspectives) rendered only a slight increase in the correlation coefficient. Interestingly, neither charge and spatial coordinate of the pHLA nor the ec pHLA yielded significant results.

**Figure 4:**
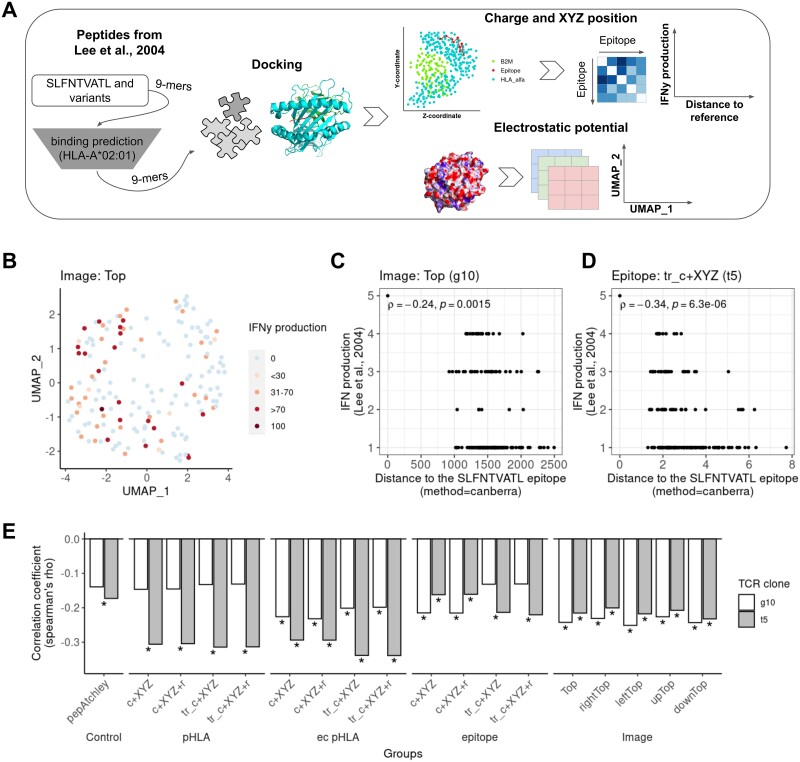
Association of TCR cross-reactivity with IFNy production. A) Workflow for obtaining the images of electrostatic potential. The SLFNTVALT epitope and its variants were used for binding predictions to HLA-A^*^02:01 to select the core nonamers to be modeled by Docktope. The electrostatic potential was calculated from the modeled structures as described in the methods and the colors of the images were divided into layers of red, green, and blue prior to manipulation and clustering. The charge and spatial position of each modeled structure were also obtained to calculate the Euclidean distances between epitopes and further used to test the correlation with the TCR reactivity. B) Electrostatic profile images of peptide-HLA complexes for the 170 selected epitopes from Lee et al. were used in UMAP generation. The images were obtained from five perspectives encompassing the HLA groove, and pixels were selected for regions of higher variability (see methods). C-D) Spearman correlation between the similarity of variant epitopes SLFNTVATL and IFNγ production. The Canberra distance was used to calculate the distance between the epitope images and gag (C), and the Euclidean distance for the distance between charge and spatial position between the epitopes and the reference SLFNTVATL (D). E) Spearman correlation coefficients for correlations of IFNγ release with combinations of charge (c), spatial position (XYZ), and radius (r) data for original epitopes or with trimmed (tr) residues at anchor positions. ^*^Spearman correlation with a p-value less than 0.05.

In a different work, Lee *et al.* [15] tested the IFNy production of two distinct T-cell clones to variants of the core peptide SLFNTVATL, an epitope derived from the Gag protein of the human immunodeficiency virus 1. We modeled SLFNTVATL and its variants to investigate the association of cross-reactivities observed *in vitro* with similarities in the structural modeling (Fig. 4A). The unsupervised clustering evidenced no particular grouping for Top pHLA images of electrostatic potential (Fig. 4B). However, weak correlations (rho = −0.24, *P* = 0.0015) were observed between the IFNy production and the distance to the original epitope (Fig. 4C). Similarly, the charge and spatial coordinates of the ec pHLA also produced weak correlations (rho = −0.34, *P* = 6.3e-06) with IFNy production (Fig. 4D). Interestingly, TCR clone t5 showed stronger correlations with IFNy production when using charge and spatial coordinates (Fig. 4E). In contrast, TCR clone g10 presented slightly stronger correlations with the IFNy production for all perspectives of electrostatic potential. Together, the data showed weak correlations between IFNy production and pHLA charge, spatial position, and electrostatic potential. Moreover, different TCRs recognizing the same antigen present distinct degrees of association with the charge and electrostatic potential.

### Electrostatic potential suggests high similarity across human epitopes

**Figure 5:**
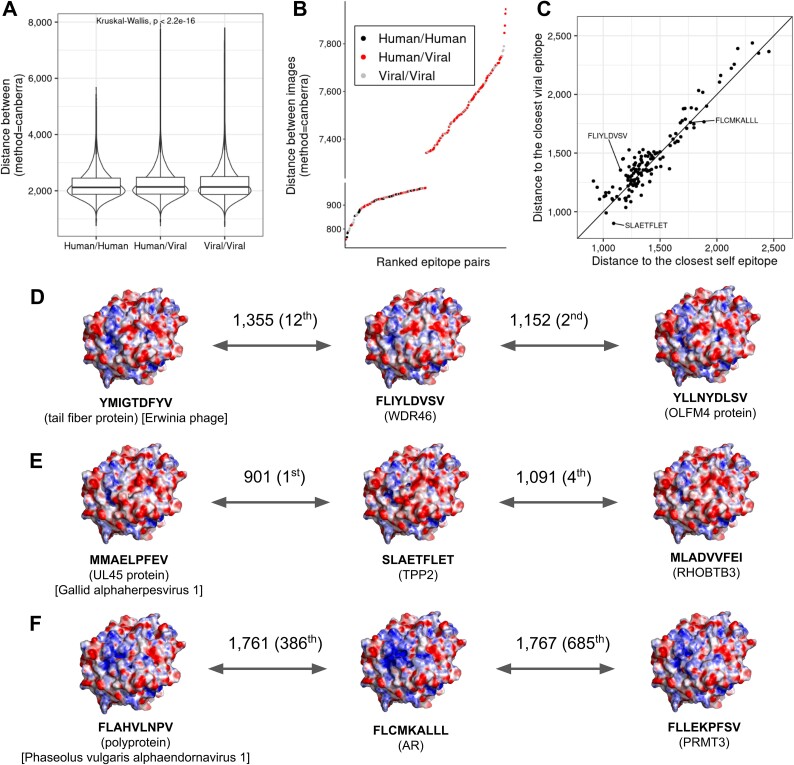
Potential cross-reactivities of tumor epitopes with viral peptides. The Euclidean distance was calculated for each unique pair of epitopes, and they were categorized into three groups based on the origin of the epitopes: Human/human, human/viral, and viral/viral. The distance was computed from the electrostatic potential obtained from the images of the top of the pHLA. B) The 300 epitopes with the highest and lowest Euclidean distance in A) were sorted according to the distance between pairs. The colors represent the group of analyzed epitope pairs. The Y-axis was intentionally broken to improve visualization. C) Euclidean distances between tumor epitopes and other epitopes of human or viral origin were calculated from the electrostatic potential of the pHLA surface, and the shortest distance for a viral (y-axis) and human (x-axis) epitope was represented. The diagonal line represents the intersection of the distance values between the two axes. The colors of the electrostatic potential represent positive (blue), neutral (white), and negative (red) charges. D-F) Electrostatic potential of the pHLA for three cancer epitopes depicted in C). The closest matches from viral and self epitopes are shown with the respective Canberra distances.

Cross-reactivity between human and pathogen peptides was previously described as the origin of some autoimmune diseases and is gaining importance in understanding responses to cancer [8, 41]. Previous works described TCR cross-reactivities to different epitopes by the average electrostatic potential [20, 22, 42]. Given the association of pHLA electrostatic potential with the TCR reactivities, we aimed to investigate similarities in pHLA electrostatic potential between self and viral epitopes. We performed a comprehensive selection of epitopes, as described in the first section, and a total of 867 878 strong binder epitopes were predicted (191 268 self and 676 824 viral). Of those, the majority was overrepresented at least once (Fig. S5), and <0.2% of self epitopes were also found in viruses (Table 1, Supplementary Data S2). Interestingly, the number of self and viral binders decreases at different rates with the increase of redundancy. Despite a number of predicted epitopes 3.5 higher, the viral binders are less redundant (Fig. S5). Disconsidering the residues from the anchor positions P2 and P9, the number of shared sequences is about three times higher for nonamers and decamers (Fig. S5C and D, Table 1). These results indicate that the majority of self epitopes is redundant, which might drive preferences for cross-reactive responses. We analysed the similarity between viral and human epitopes using the electrostatic potential of the pHLA surface. The distances from each epitope to all others were calculated from the images. Each unique pair was then classified into human/human, human/viral, or viral/viral according to the origin of the epitopes. Canberra distances calculated from the pHLA images (top perspective) showed a median of 2120 for human/human epitopes versus 2137 for human/viral and 2136 for viral/viral (Fig. 5A). Despite no difference observed in the median distribution, small distances between epitopes are more relevant for cross-reactivities. With that in mind, we explored 300 pairs of epitopes with the smallest and largest distances in the previous distribution (Fig. 5B). We observed that the concentration of human/human and viral/viral epitope pairs is higher at the bottom of the graphs (more similar), which contrasts with the higher concentration of human/viral pairs at the top.

**Table 1:** Percentage of shared self and viral epitopes.

			**Trimmed epitopes**	
**Length**	**Restricted**	**Shared**	**Restricted**	**Shared**
8	9853	12 (0.12%)	-	-
9	448 401	168 (0.037%)	437 042	590 (0.13%)
10	303 907	27 (0.0089%)	297 891	77 (0.026%)
11	105 503	7 (0.0066%)	-	-

Trimmed epitopes had anchor residues 2 and 9 removed.

As observed so far, the electrostatic potential and charge of pHLA are capable of capturing characteristics of pHLA associated with TCR cross-reactivity. In order to identify potential cross-reactivities between tumor and non-tumor epitopes, the epitopes of viral or self-origin that were more similar to the tumor epitopes were ranked. First, epitopes described in the databases as neoantigens or derived from tumor-associated antigens (TAA) were filtered, obtaining 133 epitopes. Then, distances between tumor epitopes and all viral or self epitopes modeled were calculated from the electrostatic potential of the pHLA surface. Finally, we depicted the tumor/viral and tumor/self epitope pairs with the smallest distances between them (Fig. 5C). Epitopes with greater distances have, in theory, a lower chance of cross-reactivities considering the set analysed. We observed a small shift to the left compared to the line with slope one (80 versus 53 peptides), indicating that most tumor antigens are more similar to a self antigen than to a viral one. Importantly, the wild-type peptides originating neoantigens were not considered in this analysis. This effect can be observed by the similarity between the pHLA electrostatic potentials of the neoantigen FLIYLDVSV and the two closest matches from either viral or self epitopes (Fig. 5D). In this example, the epitope YLLNYDLSV presents a lower distance (1152, 2nd closest) to the neoantigen than any of the modeled viral epitopes. In contrast, the points to the right of the line have an epitope of viral origin as the closest match. The neoantigen SLAETFLET is more closely related to the viral epitope MMAELPFEV than to the human epitope MLADVVFEI (Fig. 5E). Finally, some TAA such as the FLCMKALL present virtually the same resemblance to a self or viral epitope, since they are located on the slope curve (Fig. 5F). In this reduced universe of peptides, it was possible to observe that most neoantigens present an electrostatic profile similar to self epitopes. We confirmed this effect by resampling subsets of 100 to 500 viral and human epitopes 1000 times and observing a moderate to strong shift in the distances (Fig. S6). We divided the cancer antigens according to their resemblance to a self (CloserToHuman) or viral (CloserToViral) epitope to investigate potential distinguishing features. No striking difference was observed for single amino acid frequencies (Fig. S7A). Next, we investigated amino acid properties using Atchley factors [40], a work that consolidated several amino acid characteristics into five factors. Each peptide residue was assigned a value from the five factors and we compared the median distribution of each group (Fig. S7B). Similar values were observed for the vast majority of the positions, except for Factor 1 in Position 6 (−1.02 versus −0.591, *P* = 0.0255) and for Factor 2 in Position 8 (−0.279 versus 0.326, *P* = 0.0372). Interestingly, Factor 1 reflects the polarity of the residues both exposed and hidden [40] which corroborates the fact that groups were defined based on the electrostatic potential. Position 6 possesses more hydrophobic residues in CloserToHuman (71.2%) than CloserToViral (58.5%), which needs to be further investigated with other cancer antigens (Table 2). Identifying similarities between viral and cancer epitopes might help rank candidate epitopes for cancer vaccines. However, the choice of cross-reactive epitopes must consider the underlying similarity of cancer antigens to self epitopes that go beyond their sequence.

**Table 2:** Amino acid properties in position 6 of cancer antigens classified into more similar to a self (CloserToHuman) or viral epitope (CloserToViral).

	**CloserToHuman**	**CloserToViral**
Acid	3 (3.8%)	5 (9.4%)
Basic	2 (2.5%)	2 (3.8%)
Hydrophobic	57 (71.0%)	31 (58.0%)
Neutral	2 (2.5%)	5 (9.4%)
Polar	16 (20.0%)	10 (19.0%)
**Total**	**80**	**53**

## Discussion

We built a database of >2600 modeled pHLA to capture cross-reactivities described in the literature and to demonstrate the association of TCR reactivity to HLA-I-restricted epitopes by using pHLA electrostatic potential. Moreover, we enumerate cross-reactivities between cancer and viral epitopes that could benefit from *in vitro* testing to further confirm their value and magnitude. This work was possible thanks to previous conceptual [4, 7, 22, 43] and methodological [19, 37, 44] basis of cross-reactivity, and data from studies that evaluated the reactivity of several TCRs [8, 14, 15, 45]. The identification of cross-reactivities can help in the treatment of autoimmune diseases [14], in vaccine development planning [20, 33], and in the identification of cross-reactivities to cancer antigens [46]. However, in vitro testing of thousands of candidates for cross-reactivity is expensive and laborious. *In silico* predictions, on the other hand, are cheaper and have high scalability, overcoming the technical bottlenecks of *in vitro* testing [47]. To this end, it is imperative to better understand the structural bases that guide the TCR:pHLA interaction.

The study focused on A^*^02:01-restricted nonamers due to constraints of the modeling strategy [37] and to the relevance of this allele which is among the most frequent HLA-I [14, 48]. Most epitopes in the public databases are of viral origin, and often many CDR3 sequences are described for the same epitope. Similar responses to different viral epitopes are not an unexpected event [49], which justifies the enrichment of CDR3 sequences. In order to expand the collection of epitopes and to reduce biases associated with enriched viral sequences such as influenza, Cytomegalovirus (CMV), and Epstein-Barr virus (EBV), we modeled ligands from representative viral proteins and human proteome. This selection was based on HLA affinity which allows peptides from proteins expressed at low levels to be selected. Large-scale modeling of viral and human epitopes allowed the identification of regions with higher variability in electrostatic potential based on previously established methods [19, 22]. Our observation that cross-reactive epitopes differ from a pool of unrelated epitopes aligns with previous findings that related electrostatic potential and TCR cross-reactivity [22]. For a different set of epitopes, we observed that the charge of the amino acids directly affects the charge on the pHLA surface, as described by Antunes and collaborators. The pHLA charge and spatial position concern the entire molecule and not just the portion exposed to the TCR. The modeling reflects the lowest energy conformation, which is not necessarily the one promoting TCR engagement and activation [15, 18]. An alternative approach could combine the most likely or dynamic pHLA complexes instead of a single consensus [50]. Such a dynamic structure could increase the chances of finding associations with TCR reactivity.

We identified a weak correlation between TCR cross-reactivity among the mutated epitopes and T-cell activation [14, 15], suggesting that epitope similarity is associated with TCR engagement in this case. Some epitopes from Bulek *et al.* were capable of stimulating T-cells to produce more TNF-⍺ than the reference epitope in line with the fact that the pHLA recognized by a TCR is not necessarily the one with the greatest affinity [7, 8]. Of note, Lee *et al.* measured the frequency of responding T-cells while Bulek *et al.* measured the magnitude of response. The lack of correlation for pHLA from Bulek may suggest that PPI mutants induce fewer changes in the charge and conformation that affect the interaction with key residues of the TCR [7] or that are simply diluted in the background. The weak to moderate correlations suggest other characteristics that are not captured by the approaches used might be playing a role such as the activation threshold of T-cells [12, 51, 52], interactions that change the conformation of epitope [18], or CD8 activation [36]. Some results of epitope-lymphocyte correlations suggest that TCR recognition is less susceptible to punctual epitope changes and more dependent on pHLA interactions. A possible explanation is that epitope residues such as acid and basic more significantly affect neighboring HLA residues and that this change is essential to capture TCR reactivity in this case [22]. Finally, the distinct affinity of the tested TCRs and/or the antigen density at the cell surface could play a role in determining the associations between pHLA and epitopes [13]. In this sense, a promiscuous TCR could produce more variations in T-cell activation due to its susceptibility to activation by mutated epitopes.

Cross-reactivities between microorganisms and self epitopes were previously described [46] and might help explain clinical observations of tumor regression [53, 54] and foster the development of therapies expanding T-cells. This motivated us to study the similarity between self and viral epitopes. Our results reflected intrinsic similarities between the human and viral genomes. The fact that they are more similar to each other should be related to differences in the amino acid composition. In this sense, it is not uncommon to observe TCR recognition of similar viral sequences [49, 55]. Only a minor fraction of viral epitopes shared sequences with self epitopes. Those could be potentially advantageous for the virus, as they could escape immunological surveillance. In contrast, the engagement of TCRs reactive to self epitopes, whether associated with tumors or not, may occur after a challenge with pathological agents or vaccines [53, 56, 57] due to surpassing thresholds that restrict the reactivity to self. Reports of cross-reactivity were described during the SARS-CoV-2 pandemic such as pre-existing responses to the virus in unexposed individuals, which could indicate a prior expansion of the response following infection with another virus [58]. Other studies reported CD4 and CD8 lymphocyte responses between SARS-CoV-2 and Cytomegalovirus antigens [55], and responses between different variants of SARS-CoV-2 [59]. Altogether, an investigation of the molecular basis behind cross-reactivity may bring therapeutic benefits to cancer patients and aid in vaccine development strategies.

Cross-reactivities involving public TCRs may be more valuable for therapeutic approaches as their frequencies are higher in the population. However, the characterization of public TCRs and an individual’s TCR repertoire requires a large number of TCR sequences [60]. Another important factor in TCR cross-reactivity is related to the T lymphocyte subtypes involved in the response. The repertoires of CD4^+^ T and CD8^+^ T lymphocytes have a small overlap due to the characteristics of the HLA classes they recognize [61]. Unlikely, cross-reactive TCRs in regulatory T-cells should produce tolerogenic responses compared to conventional CD4^+^ T-cells or CD8^+^ T-cells. The cross-response between a *Clostridium asparagiforme* epitope and PPI described by Cole *et al.* [8] is an example of how a non-self antigen could induce tolerance breakdown. Besides, possible cross-reactivity between a tumor antigen and a non-self antigen could be of clinical interest. In this case, the breakdown of tolerance could induce a response to the tumor antigen. We observed that only a minority of the viral epitopes analysed might cross-react with self epitopes, hindering the search for clinical cross-reactivities.

## Conclusion

In this study, we developed a comprehensive database of modeled epitopes, relating electrostatic potential to TCR reactivity and cross-reactivity. We identified distinct features in viral pHLA closely related to cancer epitopes, and listed potential cross-reactive epitopes. Our findings underscore the challenge of identifying viral antigens that mimic cancer epitopes, which has significant implications for enhancing anti-tumor responses. Besides, the thousands of modeled self and viral pHLA might serve in future works for identifying pathogen escape variants and predicting potential autoimmunity.

**Key Points**

- We generated a database of viral and human epitopes to study cross-reactivity;
- Cross-reactive CDR3 sequences from public databases recognize epitopes with similar electrostatic potential, charge, and spatial location.
- We observed a negative correlation between T-cell activation and epitope dissimilarity, as captured by electrostatic potential.
- We enumerated potential cross-reactivities with cancer epitopes and highlighted challenges in their identification for therapeutic use.

## Supplementary Material

Figure_S1_bbaf012

Figure_S2_bbaf012

Figure_S3_bbaf012

Figure_S4_bbaf012

Figure_S5_bbaf012

Figure_S6_bbaf012

Figure_S7_bbaf012

Table_S1_bbaf012

Supplementary_Data_1_bbaf012

Supplementary_Data_2_bbaf012

## Data Availability

Data such as structural models and code for this analysis are available at: https://github.com/marcopretti/Pretti_BiB_2025.
