# Correction to: Unveiling cross-reactivity: implications for immune response modulation in cancer

**DOI:** 10.1093/bib/bbaf068

**Published:** 2025-01-06

This is a correction to: Marco Antônio M Pretti, Gustavo Fioravanti Vieira, Mariana Boroni, Martín H Bonamino, Unveiling cross-reactivity: implications for immune response modulation in cancer, *Briefings in Bioinformatics*, Volume 26, Issue 1, January 2025, https://doi.org/10.1093/bib/bbaf012

In the originally published version of the manuscript, a second affiliation was missed for the last co-author. This is now added in the article's affiliation list, and the enumeration in the author list altered respectively.

