# Supplementary figures and images for: Unveiling cross-reactivity: implications for immune response modulation in cancer
Source: Brief Bioinform. 2025 Jan 20;26(1):bbaf012. doi: 10.1093/bib/bbaf012 (PMC11744606; doi:10.1093/bib/bbaf012)

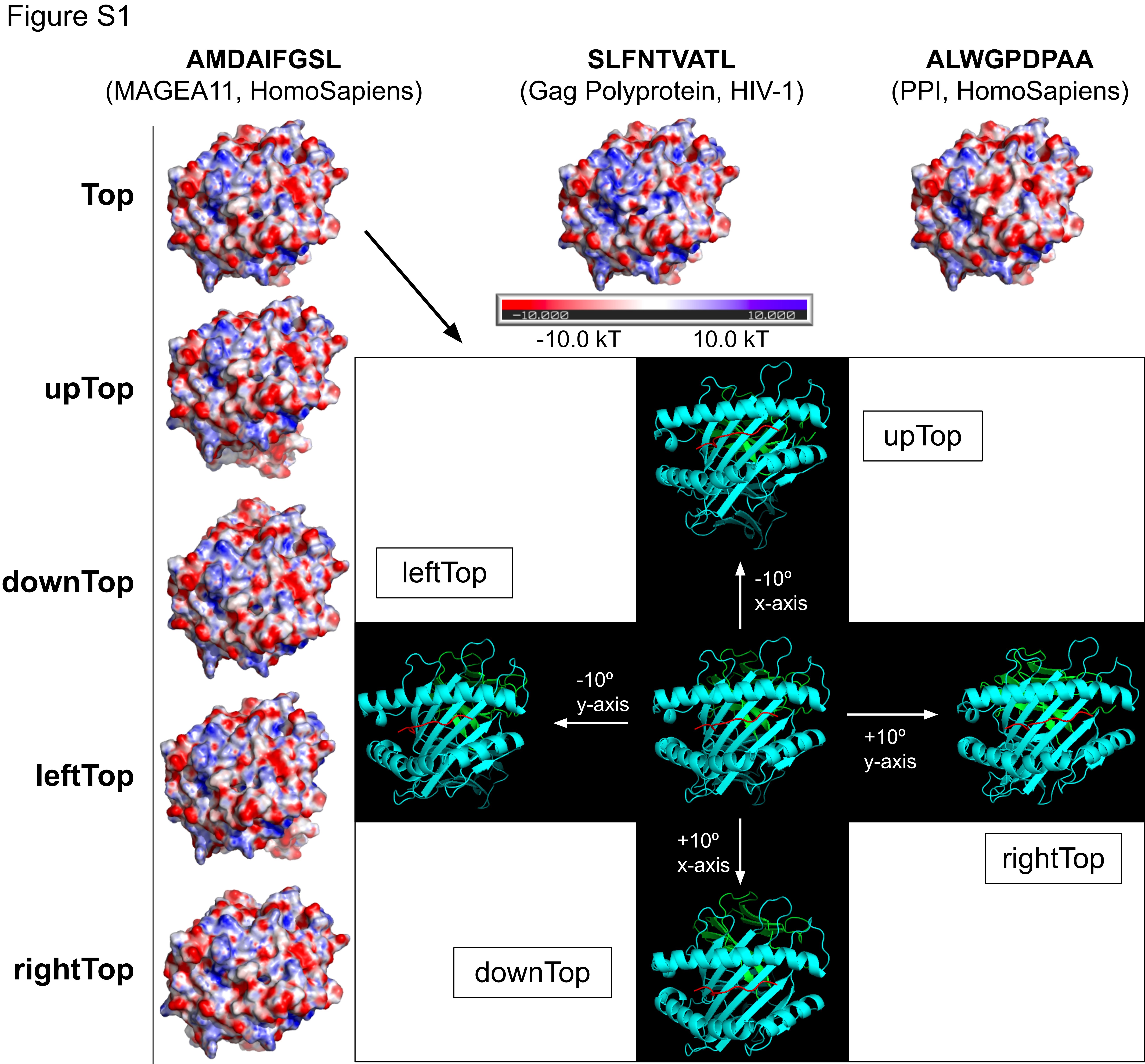

Supplement: Figure_S1_bbaf012 [file figure_s1_bbaf012.jpeg]

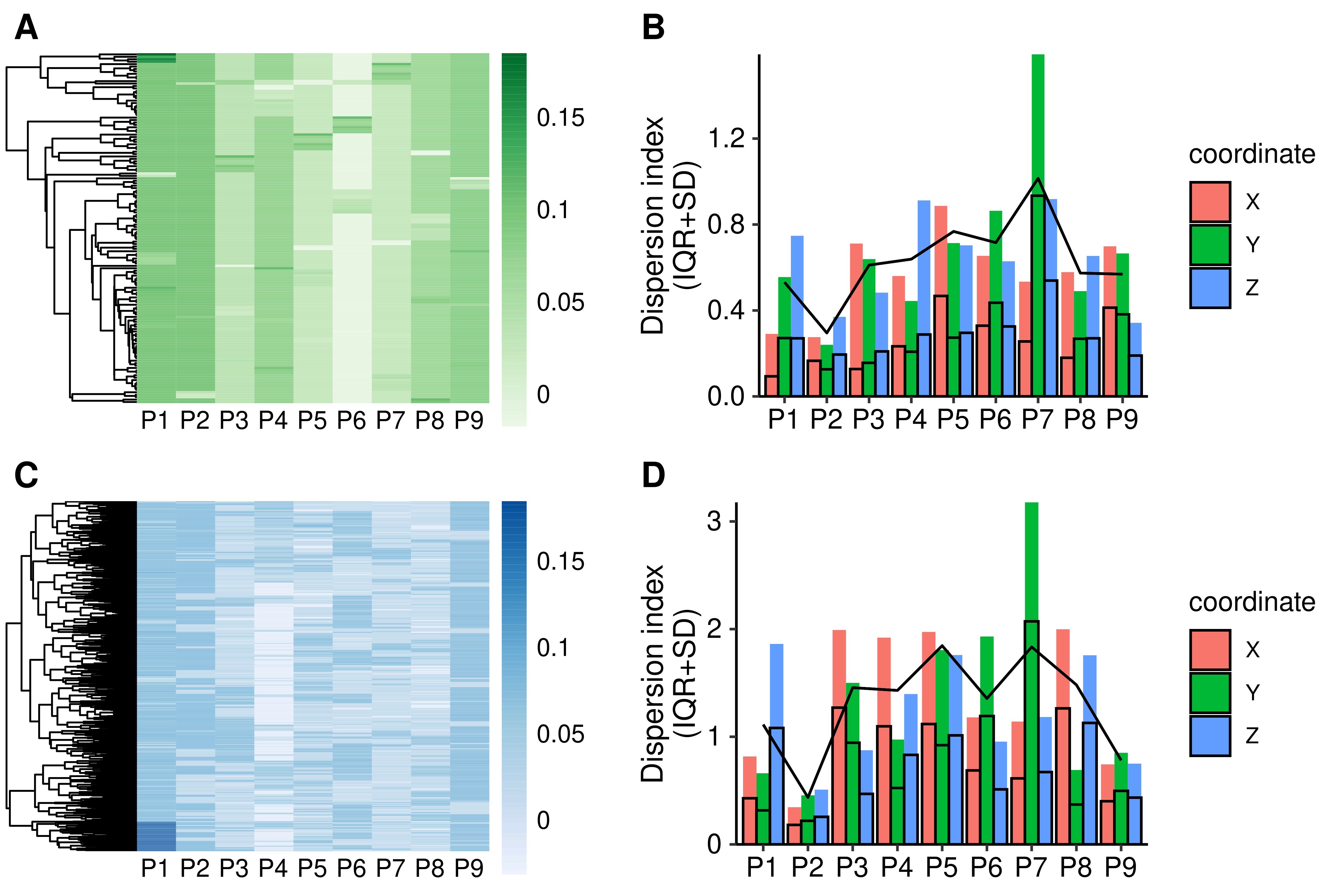

Supplement: Figure_S2_bbaf012 [file figure_s2_bbaf012.jpeg]

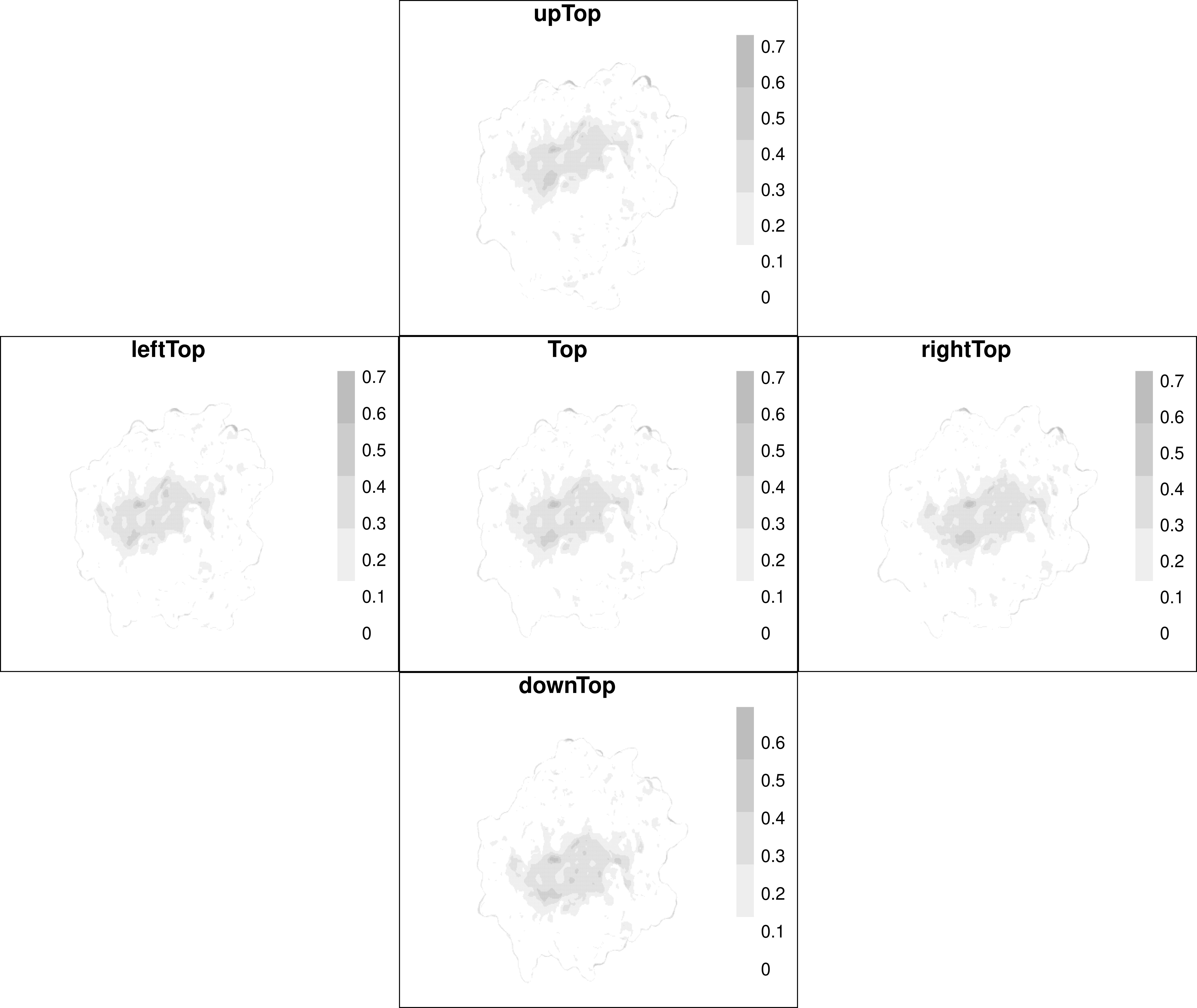

Supplement: Figure_S3_bbaf012 [file figure_s3_bbaf012.jpeg]

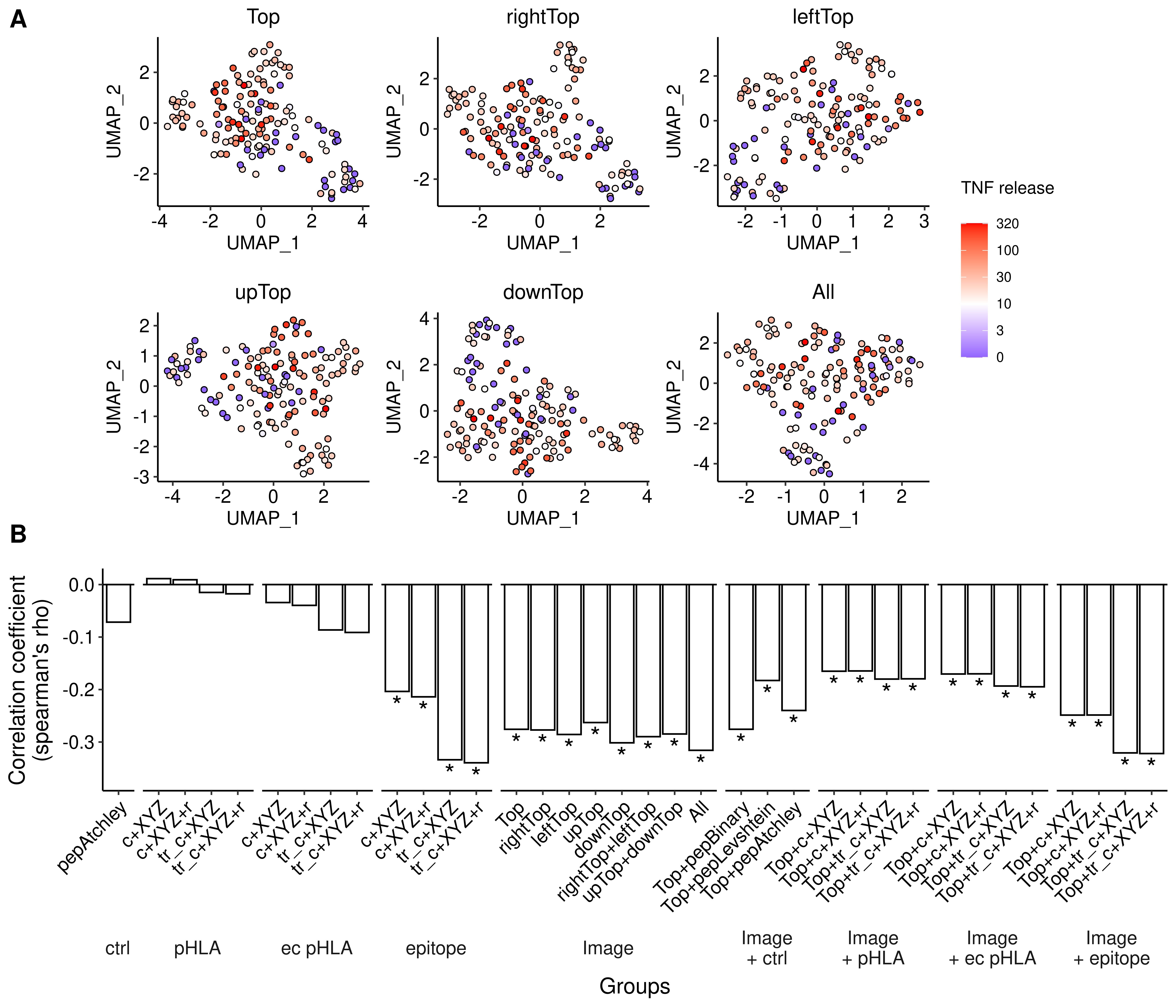

Supplement: Figure_S4_bbaf012 [file figure_s4_bbaf012.jpeg]

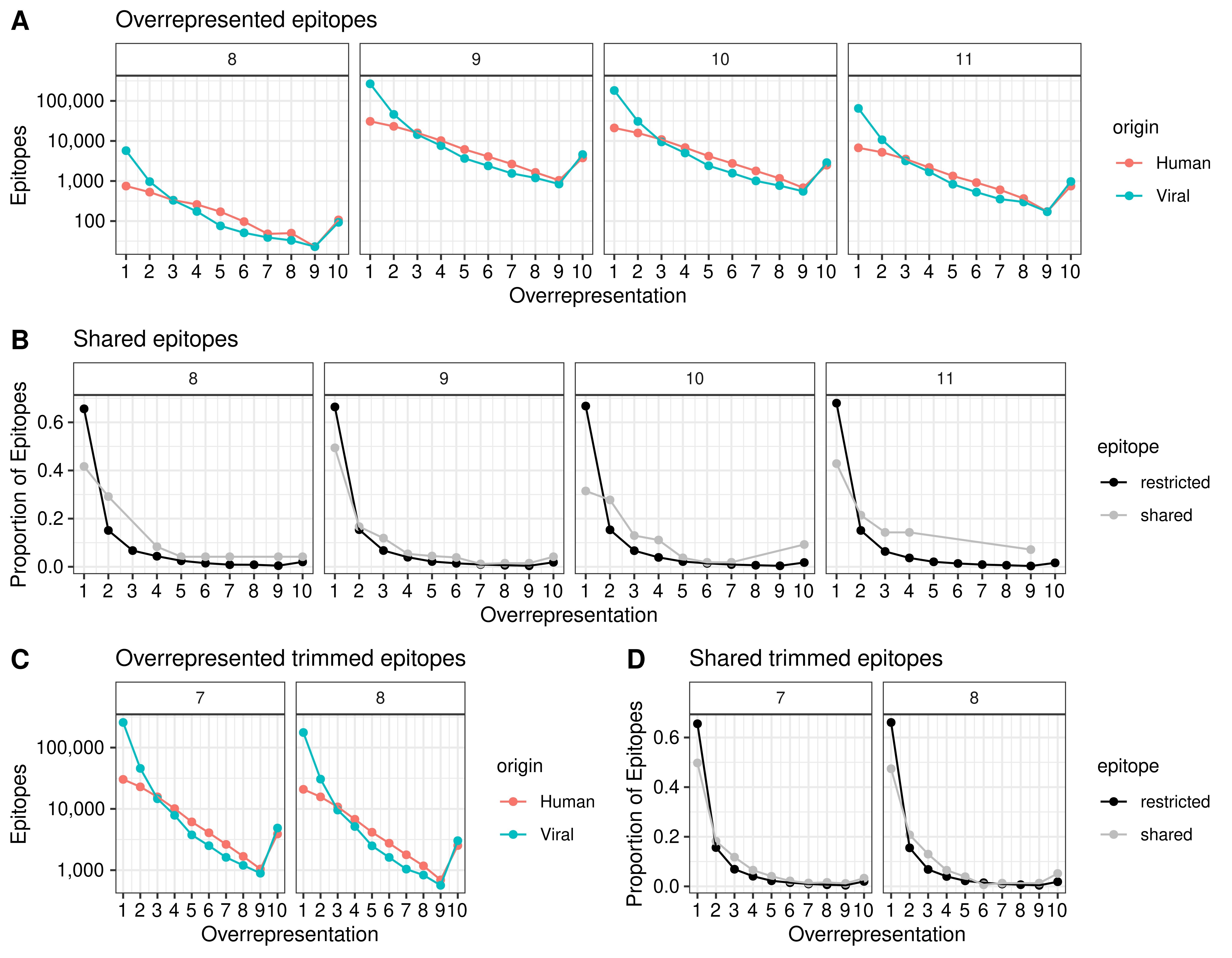

Supplement: Figure_S5_bbaf012 [file figure_s5_bbaf012.jpeg]

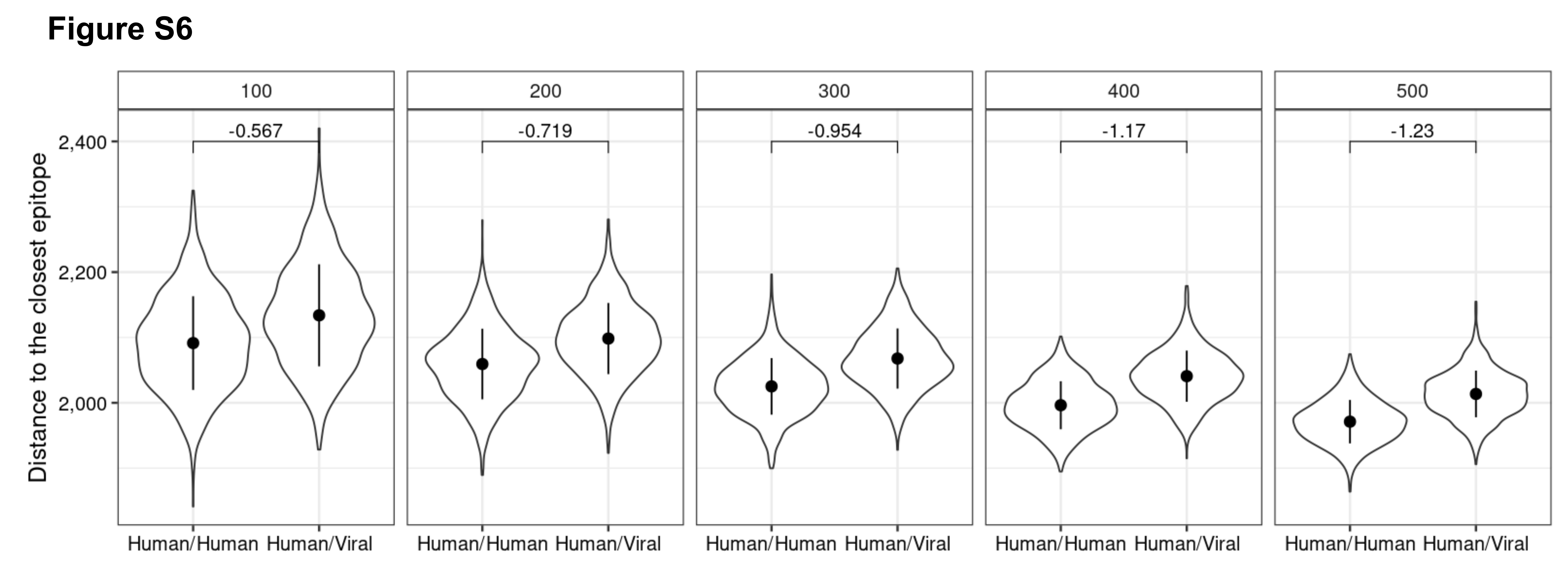

Supplement: Figure_S6_bbaf012 [file figure_s6_bbaf012.jpeg]

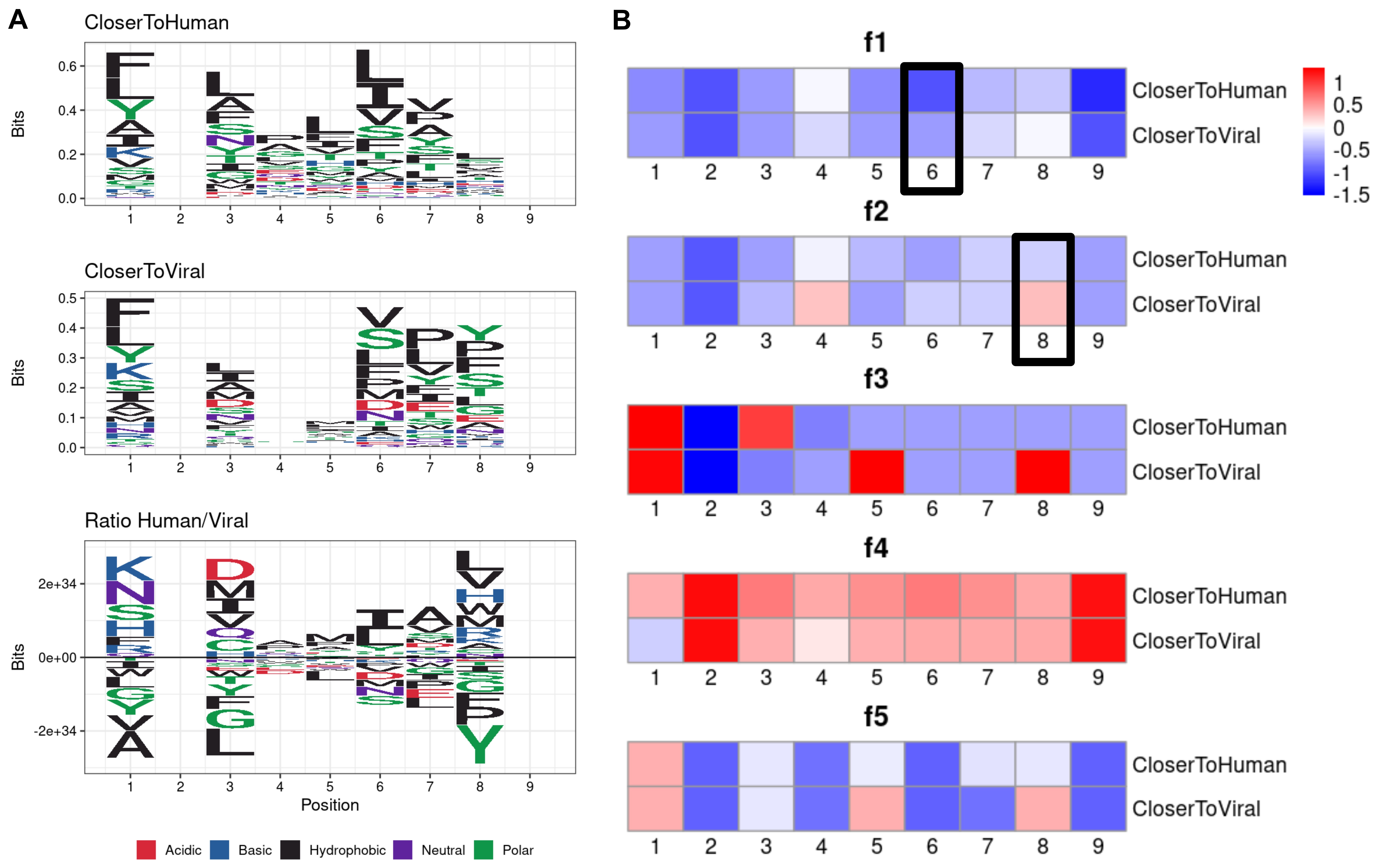

Supplement: Figure_S7_bbaf012 [file figure_s7_bbaf012.jpeg]
