# Supplementary material for: Unveiling cross-reactivity: implications for immune response modulation in cancer
Source: Brief Bioinform. 2025 Jan 20;26(1):bbaf012. doi: 10.1093/bib/bbaf012 (PMC11744606; doi:10.1093/bib/bbaf012)
Supplement: Table_S1_bbaf012 [file table_s1_bbaf012.doc]

Table S1. Distribution of the modeled epitopes

| **Reference** | **Modeled Epitopes** |
| --- | --- |
| GRCh38 | 989 |
| NCBIViral | 987 |
| McPAS | 200 |
| Lee2004 | 168 |
| Bulek2012 | 143 |
| VDJdb | 122 |
| McPAS, VDJdb | 10 |
| McPAS, TBAdb, VDJdb | 7 |
| Lee2004, VDJdb | 2 |
| Bulek2012, McPAS | 1 |
| GRCh38, McPAS | 1 |
| NCBIViral, VDJdb | 1 |
